# A matter of emphasis: Linguistic stress habits modulate serial recall

**DOI:** 10.3758/s13421-014-0466-2

**Published:** 2014-10-04

**Authors:** John C. Taylor, Bill Macken, Dylan M. Jones

**Affiliations:** School of Psychology, Cardiff University, Cardiff, CF10 3AT UK

**Keywords:** Short-term memory, Serial recall, Speech production, Articulation

## Abstract

Models of short-term memory for sequential information rely on item-level, feature-based descriptions to account for errors in serial recall. Transposition errors within alternating similar/dissimilar letter sequences derive from interactions between overlapping features. However, in two experiments, we demonstrated that the characteristics of the sequence are what determine the fates of items, rather than the properties ascribed to the items themselves. Performance in alternating sequences is determined by the way that the sequences themselves induce particular prosodic rehearsal patterns, and not by the nature of the items per se. In a serial recall task, the shapes of the canonical “saw-tooth” serial position curves and transposition error probabilities at successive input–output distances were modulated by subvocal rehearsal strategies, despite all item-based parameters being held constant. We replicated this finding using nonalternating lists, thus demonstrating that transpositions are substantially influenced by prosodic features—such as stress—that emerge during subvocal rehearsal.

Phonological similarity is perhaps the most noteworthy phenomenon in the canon of verbal short-term memory (vSTM) research. Its effects are very widely studied (e.g., Baddeley, 1966, 1968; Conrad, 1964; Farrell, 2006; Farrell & Lewandowsky, 2003; Henson, Norris, Page, & Baddeley, 1996; Wickelgren, 1964), and the task of accounting for the cost to recall performance of phonological similarity is fundamental to any model of vSTM. Of the several empirical manifestations of phonological similarity, the most demanding theoretically is the particular pattern of serial recall performance in sequences containing alternations of similar- and dissimilar-sounding items (e.g., the letter sounds *b*, *f*, *p*, *k*, *c*, *l*, . . . , or *f*, *b*, *k*, *c*, *l*, . . .), relative to homogeneous sequences comprising only either similar- or dissimilar-sounding items (see, e.g., Baddeley, Papagno, & Norris, 1991; Farrell & Lewandowsky, 2002; Henson et al., 1996). Several features of this comparison are striking, including the shapes of the serial position curves and the patterns of transposition errors. Lists in which similar items are alternated with dissimilar items have a saw-tooth-shaped serial position curve, whose most striking feature is that the level of performance associated with each item remains the same, regardless of the context (homogeneous or alternating). Another significant feature of the pattern of results is the pattern of transposition errors. Commonly, in homogeneous lists of similar- or dissimilar-sounding items, transposition errors are most likely over short input–output distances (the constraint of locality). Moreover in alternating lists, transpositions are more likely at input–output distances of *N*_(±2)_ than at *N*_(±1)_, as is found in homogeneous lists. Since similar items are more likely to be transposed than dissimilar items (the constraint of similarity), *N*_(±2)_ errors have been proposed as the vehicle through which the characteristic “saw-tooth” serial position curves are made manifest (Henson, 1998; Henson et al., 1996). By this means, the likelihood of any two items being transposed during serial recall is co-determined by item-to-item similarity and by the number of intervening items within a to-be-recalled sequence.

Conventional wisdom holds responsible the action of item-level representations for these and other effects of phonological similarity. Errors at storage, encoding, or retrieval are held to result predominantly from overlapping phonological features (e.g., Farrell & Lewandowsky, 2004; Page, Madge, Cumming, & Norris, 2007). Such accounts have had some success in explaining how an item’s fate is independent of (homogeneous/alternating-list) context. Nevertheless, a considerable body of recent research has indicated that such mechanisms provide an incomplete account of these phenomena. More generally, in relation to phonological similarity and its role in serial recall, evidence is beginning to suggest that similarity effects can be attributed to domain-general auditory perceptual and motor control processes, rather than to specifically phonological ones (e.g., Jones, Hughes, & Macken, 2006; Jones, Macken, & Nicholls, 2004; Maidment & Macken, 2012). From this perspective, revisiting the broad characteristics of serial recall performance with the especially challenging case of alternating lists is timely. This article is devoted to exploring the possibility that the domain-general approach can be applied to the patterns of errors in homogeneous and alternating lists.

Conventionally, and at its simplest, the contrast between alternating and homogeneous lists can be conceived as the composite of individual-item interactions, independent of the context in which the items are placed: That is, an item (e.g., a word or letter sound) will be just as memorable whether alongside similar or dissimilar items. This points to an important common assumption of most accounts of serial recall and an implicit aspect of the concept of phonological similarity itself—namely, that the unit of explanatory currency is the operationally defined item, characterized principally by its phonological composition. The hegemony of the item and its phonological constituents is made explicit in a wide range of modeling approaches that have also been applied to the broader analysis of vSTM (e.g., Brown, Preece, & Hulme, 2000; Burgess & Hitch, 1999; Farrell & Lewandowsky, 2002; Henson, 1998; Nairne, 1990; Page & Norris, 1998). One consequence of this item-based view is that the roles of perceptual and effector components—those most directly implicated in the organization and implementation of temporally extended sequences—are accorded subordinate, sometimes epiphenomenal status, so as to be regarded as being peripheral to the core “cognitive” activity, so that their impact is restricted to processes either preceding or following, but emphatically partitioned from, the modular processing primitive that is the memory system.

Increasingly, rather than seeing such item-focused memory processes as the basis underpinning performance, emerging accounts seek to relate the mechanisms of vSTM to more general linguistic ones (e.g., Acheson & MacDonald, 2009; MacDonald & Christiansen, 2002; Martin & Saffran, 1997), including those involved in the organization of speech perception and production (e.g., Hickok, 2009; Hickok & Poeppel, 2004; Scott, McGettigan, & Eisner, 2009). Here, memory is embodied within the sensory–motor system, and this embodiment is consequential for memory performance. Of course, the classical cognitivist approach to linguistic mechanisms (e.g., Chomsky & Halle, 1968), like those cognitivist approaches to short-term memory, incorporate primordial, discrete phonological segments as the essential building blocks. However, more recent usage-based accounts have shown that the hallmarks of linguistic behavior can emerge from domain-general processes that operate over extended and continuous perceptual and motor representations (e.g., Goldstein, Pouplier, Chen, Saltzman, & Byrd, 2007; Port & Leary, 2005). From this usage-based perspective, the primordial unit is not the item per se, made manifest through its phonological character. Rather, the status of those smaller segments is derived from superordinate entities—namely, those of utterances, phrases, or lexical units (see, e.g., Bybee, 2010; Norris, McQueen, & Cutler, 2003).

The chief implication of applying this approach to vSTM is a shift in the locus of explanation from the item to the sequence. The fate of nominal items is not to be determined primarily by their inherent characteristics, but rather is an emergent feature of the sequential structure in which they are embedded. Indeed, it is methodologically relevant that the stimuli typically selected for use in vSTM experiments—guided by theoretical assumptions regarding the discrete phonological nature of vSTM—would tend to restrict severely any influence of linguistic processes. The to-be-remembered sequences are commonly novel concatenations of single-item utterances, lacking both the articulatory familiarity and coarticulatory fluency of natural speech.

If such a shift in conceptual frame—toward the linguistic, embodied, continuous, and articulatory—is to be sustained, its tenets must be shown to be effective in settings that have been used to validate the classical item-based view. This challenge is taken up here using the alternating-lists setting, by modulating similarity effects through manipulation of higher-order properties of verbal sequences. If vSTM is to be attributed to processes occurring within the language system, then functional aspects of the latter must be discernible in the former. In this respect, studies of transposition errors in spoken language offer strong evidence that sequence-level properties play a determining role in defining error patterns in speech. For example, although similarity in phonemic features is one influence on whether or not two sounds will interact to produce an error (e.g., consonants that share voicing or manner are more likely to transpose than those that do not; MacKay, 1970), the likelihood of those errors is still a function of characteristics of the syllable, the word, or the phrase, rather than of the features of the subordinate segment per se. So, onset clusters transpose relatively frequently, with elements of the rime transposing much less so (e.g., Shattuck-Hufnagel, 1983), and when segments do transpose, they do not do so randomly, but rather tend to move to parallel locations in the target. Similarly, the higher-level structure of stress within an utterance also determines the likelihood of errors in speech output, with stressed segments being more error prone (although this result might be only apparent, since unstressed errors may be less detectable, and therefore transcribed less reliably than stressed ones) and, again, segments sharing a stress value being more likely to interact (e.g., Shattuck-Hufnagel, 1992). Importantly, these determinants of errors in producing sequences of speech sounds transcend the segmental particulars and belong rather to the sequence than to the segment. Indeed, analysis of a corpus of experimentally induced speech errors led Shattuck-Hufnagel (1992) to conclude that “the mere presence of phonologically similar segments in the target words of an utterance, without other structural factors in common, is only a weak determinant of segmental interaction patterns” (p. 223). If, as we claim, verbal memory is parasitic on the spoken language system, then it follows that the type of transposition errors that occur within alternating lists will be dependent upon sequence-level prosodic structures commonly encountered in natural language such as metrical stress, rather than being solely an outcome of the phonological character of the nominal items.

The experiments reported here addressed this question directly by examining how the prosodic structure of visually presented verbal sequences modulates the likelihood of transpositions within those sequences. We proposed an alternative account of the typical patterns of transposition errors and serial recall curves found within alternating similar/dissimilar sequences. Rather than focusing solely on the phonological status of the items per se, we proposed that the phonological structure of the sequence as a whole would tend to induce a particular sequence-level prosodic structure, which in turn would interact with the nominal items to determine their fates. In particular, our suggestion was that the alternating structure of the DSDSDS (where “D” indicates a dissimilar item and “S” a similar one) sequence would afford a segmentation and a prosodic structure in which, not only would the alternating similar items share phonological features, but critically, they would share a stress value and relative position within each segment of the sequence (e.g., D-s, D-s, D-s, where capitalization indicates stress value and segmentation is indicated by commas), thus rendering them especially vulnerable to transposition. To the extent that this sequence-level structure of stress and segmentation was altered, then so, too, should the pattern of errors associated with those items.

We used a variant of the serial recall methodology reported by Henson et al. (1996) in which six-item to-be-recalled sequences of letters were presented in the visual modality. Our critical manipulation was the use of a simple visual cue, informing participants of the need to subvocally rehearse the sequences using a speech rhythm based on either pairs or triplets, as a means of inducing divergent patterns of metrical stress during rehearsal.

Few previous studies have investigated the effect of metrical stress on serial recall; however, these studies have typically focused on the acoustic correlates of stress—how these are preserved between encoding and output, and their effectiveness as markers of perceptual grouping boundaries (Morgan, Edwards, & Wheeldon, 2014; Parmentier & Maybery, 2008; Reeves, Schmauder, & Morris, 2000). In contrast, in the present work we sought to exploit speech habits during retention/rehearsal. Accordingly, we employed visual list format as a cue to rehearse in either pairs of triplets, but *no* specific instructions were given regarding prosodic structure. Such an unconstrained remit would in principle allow for idiosyncratic rehearsal strategies. However, if our general theoretical position is correct, we might reasonably expect participants to spontaneously adopt rehearsal patterns to track the dominant patterns of English speech—which are typically stress-initial (e.g., Shattuck-Hufnagel, 1992). We therefore predicted stable patterns of stress at output, characterized by *trochaic* (STRONG–weak) pairs and either *cretic* (STRONG–weak–STRONG) or *dactyl* (STRONG–weak–weak) triplets. In the latter case, despite still sharing phonological features, no two similar-sounding items would share both stress and relative position values (e.g., D-s-D, S-d-S or D-s-d, S-d-s). As such, the patterns of stress that would emerge would do so as the result of learned speech habits, not as a result of perceptual grouping per se. The consequences of such manipulations for serial recall and error patterns would address the tenability of an account of vSTM that elevates the sequence above the item in the explanatory hierarchy.

In Experiment 1, the sequences comprised alternating phonologically similar and dissimilar items. Previous work (e.g., Farrell & Lewandowsky, 2003; Henson et al., 1996) has shown that transposition errors should show an *N*_(±2)_ structure, consistent with the principle of similarity (Henson, 1998) and that these errors should quantitatively underpin saw-tooth serial position curves. Crucially, if errors are susceptible to influence by sequence-level prosodic processes, we predicted that rehearsing in triplets would increase the *N*_(±1)_ error rate relative to rehearsing in pairs, since the prosodic consequences of pairs rehearsal would impose an alternating structure of articulatory similarity across the sequence.

Just how the patterns of stress interact with other articulatory factors (such as those conventionally understood as phonological) will depend on the order of alternation within the sequence (SD vs. DS). Sequences that differ in alternation order but are subject to identical rehearsal would not, therefore, be expected to give rise to mirror-image serial position curves. For example, in alternating sequences involving pairs rehearsal, transposition of phonologically similar items may be rendered especially likely due to their position within the overall prosodic structure of the sequence (i.e., they are not only similar but share a common stress). Inducing a triplet form of rehearsal would accord each similar item a distinct stress value and syllable position, which in turn would decide the likelihood of its retrieval.

In Experiment 2, we applied the same manipulation not to alternating lists, but to homogeneous lists of similar- and dissimilar-sounding letters. In the absence of an alternating pattern of phonological features, an item-based account predicts that errors would follow an *N*_(±1)_ structure, with transpositions being constrained by locality (e.g., Henson et al., 1996). However, if as we predicted, transpositions are influenced by the divergent articulatory demands of prosodic stress during rehearsal in pairs and triplets, pairs rehearsal would again invoke more *N*_(±2)_ errors and fewer *N*_(±1)_ errors, relative to triplets.

Note that we do not predict that a saw-tooth serial position curve will result from this modulation. Although we do predict that within each subset of next-nearest neighbors (odd- vs. even-numbered positions) the pattern of stress will increase the likelihood of transposition, it is not clear whether this would lead to transpositions being more or less likely between odd than between even positions.

## Experiment 1

### Method

#### Participants

A group of 16 participants (12 female, four male; mean age 20 years) were recruited from the Cardiff University Human Participant Panel. Informed consent was obtained in accordance with Cardiff University, School of Psychology ethics procedures.

#### Materials

**Fig. 1:**
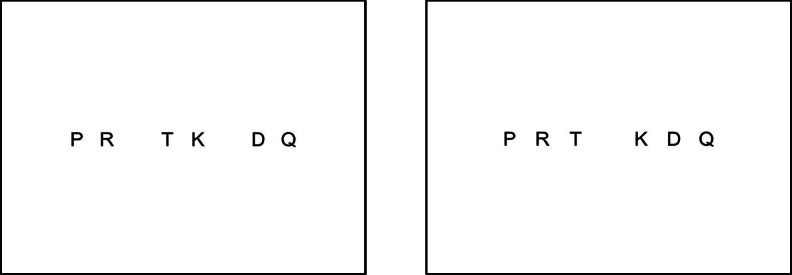
Examples of stimuli employed in Experiment 1: An SD_1_ sequence in pairs (left) and triplets (right) groupings

The stimuli comprised two sets of six consonants. The letter sets were either similar (S) sounding—*P*, *G*, *B*, *V*, *T*, *D*—or dissimilar (D) sounding—*M*, *R*, *H*, *Q*, *Y*, *K*. Three items were selected from each set and recombined to produce two heterogeneous sets, each containing three similar-sounding and three dissimilar-sounding items: *P*, *D*, *T*, *R*, *K*, *Q* and *G*, *B*, *V*, *H*, *M*, *Y*, respectively. Four complementary alternating sequences were prepared from the two sets, obeying the order conventions SDSDSD and DSDSDS; these were designated SD_1_, SD_2_, DS_1_, and DS_2_. Thirty-six unique sequence orders were assembled for every set, preserving the alternating sequence structure. Two visual displays of each sequence were prepared, conforming to either a pairs or a triplets spatial grouping. The item spacing was such that the total visual angle of each sequence was held constant across the two groupings (Fig. 1).

#### Design

A 2 (grouping structure: pairs, triplets) × 2 (similarity structure: SD, DS) within-participants design was employed. Each participant performed two experimental runs (one of each grouping structure). Each run comprised four blocks (SD_1_, SD_2_, DS_1_, and DS_2_), each of which comprised 16 sequence permutations, sampled randomly without replacement, drawn respectively from each of the four lists. Block orders were constructed, with the constraint that each half of each run comprised one SD and one DS block. Furthermore, within each run-half, SD_1_ blocks were paired together with DS_2_ blocks, whilst SD_2_ blocks were paired with DS_1_. This gave a total of eight block orders, crossed with two run orders, and each of these 16 permutations was tested once across participants.

#### Procedure

Participants were seated in front of a computer monitor and keyboard, in a sound-attenuating booth. Stimuli were presented visually on the computer monitor, and spoken recall responses were recorded via a condenser microphone positioned within the booth. All stimulus presentation and response capture was performed using MATLAB 7.11 (MathWorks, Nantick, NJ) running the Psychophysics Toolbox (Brainard, 1997; Pelli, 1997).

At the beginning of each block, the six to-be-remembered items for the block were displayed simultaneously, evenly spaced, and in an order not seen in any of the trials. Participants were instructed to familiarize themselves with the items, in order to minimize the likelihood of omission or intrusion errors. The first trial was then initiated by means of a keypress when the participant was ready. Participants were alerted to the start of each trial by the appearance of a fixation point on the screen. The six-item array was then presented on screen for duration of 1 s. At the offset of the array, participants were instructed to covertly rehearse the presented sequence. After an interval of 10 s, a red, rightward-pointing triangle was displayed on screen, cueing the participant to recall orally the six items in the correct order. Prior to its commencement, participants were made aware that a trial would consist exclusively of the items displayed at the start of the block and that no items would repeat within a trial. However, if they were unable to remember the identity of an item, they should replace it with the word “blank.” This ensured that every trial response comprised six items, preventing any ambiguity to serial recall scoring that omitted items would incur. The participant terminated each trial manually by means of a keypress. Participants were then instructed to initiate the next trial. This cycle continued until the end of the block (16 trials), at which point a fresh array of items was presented for familiarization. Oral responses were then transcribed by the experimenter and scored according to strict serial order criteria.

### Results

The serial recall data were considered in two complementary dimensions. First, a quantitative assessment of the serial position curves was undertaken. A second analysis concerned the pattern of transposition errors made at each output position.

#### Stress

In order to confirm that the format of display presentation did indeed promote different stress patterns, the recorded oral outputs for each of the four combinations of rehearsal and similarity structure were sampled representatively, such that a total of 16 trials were analyzed for each participant, equally divided across the four similarity/rehearsal conditions and selected equally often from each block, in order to minimize order effects.

The test batches thus contained a mixture of fully correct sequences and sequences containing one or more errors. An independent listener (a graduate student volunteer from within the School of Psychology), who was naïve to the purpose of the experiment, coded for stress at each serial position 16 examples of each combination of similarity and grouping structure. The stress at each serial position was then expressed as a proportion score, giving a probabilistic description of the emergent stress pattern in each grouping and similarity condition. Test–retest reliability was determined by performing the analysis twice, with a gap of one week between sessions. Both sessions were performed by the same listener, and presentation order was scrambled between sessions.

**Fig. 2:**
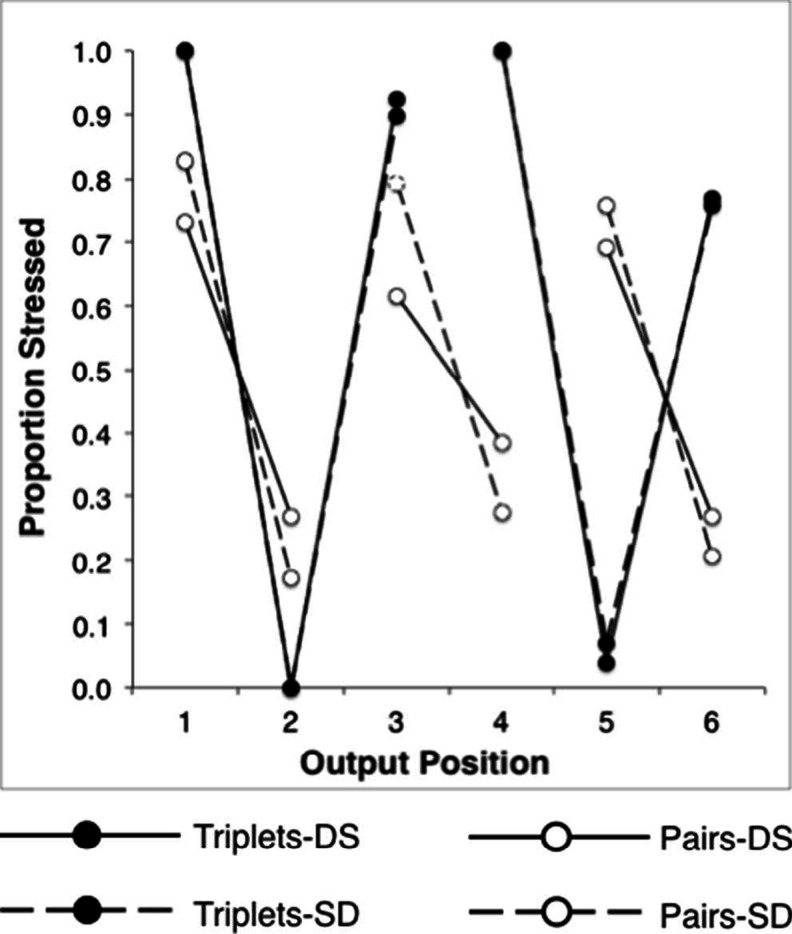
Prosodic stress probabilities for six-item alternating sequences, conforming to DS (solid lines) and SD (broken lines) structures, during recall under pairs (open circles) and triplets (closed circles) rehearsal

The test–retest scores were highly correlated, *r*(24) = .97, *p* < .001 (two-tailed) indicating a reliable stress classification. Consistent with our hypothesis, the two rehearsal methods gave rise to distinct stress patterns (Fig. 2). These were qualitatively similar, irrespective of similarity structure. Recall under the pairs rehearsal of both SD and DS structures was trochaic (i.e., STRONG–weak). The same stimuli under triplets rehearsal were recalled with a cretic meter (i.e., STRONG–weak–STRONG). Note that this was not merely an artifact of dividing the stress markers into twos and threes, since the stress probabilities across items can be seen to invert in the second half of the sequences for triplets, relative to pairs. A further interesting feature of these data is the reliability with which each rehearsal strategy gave rise to its characteristic stress pattern. Triplets were associated with an almost perfect cretic meter, independent of similarity structure, whilst the pairs data suggest greater uncertainty in stress placement, with DS being less well defined than SD.

#### Serial position curves

**Fig. 3:**
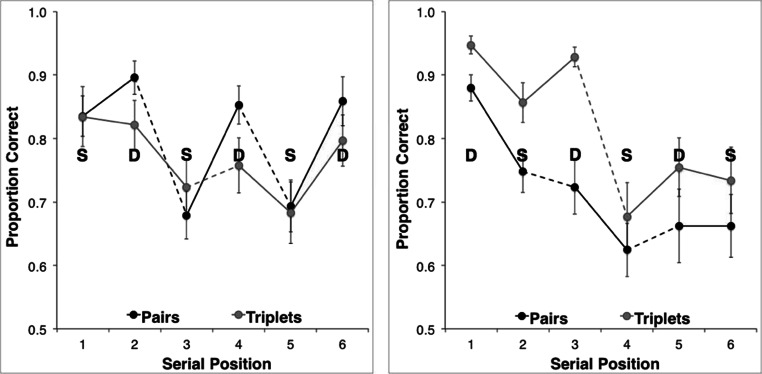
Serial position plots depicting mean (*N* = 16) performance obtained for six-item, alternating SD (left) and DS (right) sequences under the two rehearsal strategies. Error bars denote standard errors

Correct-in-position proportion-correct scores were plotted separately for each grouping strategy condition (Fig. 3). Performance for both of the similarity structures approximated to a saw-tooth pattern under both rehearsal conditions. More generally (and consistent with previous reports), recall for similar-sounding items can be seen to be inferior to that for dissimilar-sounding items. However, the saw-tooth patterns are not symmetrical for SD and DS formats *within* each rehearsal condition, nor do identical structures appear to drive equivalent performance *between* the two conditions. These two asymmetries emerge from sequences that are—item by item—identical, and they address complementary aspects of our hypothesis. Asymmetry within a grouping condition implies that perceptual and/or prosodic organizational factors interact differently with each similarity structure (i.e., that SD and DS structures are nonequivalent). Meanwhile, the effects between grouping conditions suggest that transposition likelihoods are also modulated by the particular syllabic and prosodic structures imposed by rehearsal (cf. Shattuck-Hufnagel, 1992).

In order to fully characterize the quantitative performance under each grouping condition, our data were subjected to a 2 (rehearsal: pairs, triplets) × 2 (similarity structure: SD, DS) × 6 (serial position) repeated measures analysis of variance (ANOVA). To counter potential floor and ceiling effects inherent in proportion data, the raw proportion scores were subjected to an arcsine transformation prior to statistical analysis.

Our hypothesis made no specific predictions regarding the significance of main effects, but we report them here for completeness. Consistent with previous reports (Farrell & Lewandowsky, 2003; Henson et al., 1996), the main effect of serial position was significant, *F*(5, 75) = 24.31, *p* < .001 (*η*_*p*_^*2*^ = .62). The main effects of similarity structure and rehearsal strategy both were not significant (*p*s *>* .05).

Commensurate with our core prediction that rehearsal would differentially (and as a function of similarity structure) modulate task performance, the interactions between rehearsal strategy and both serial position and similarity structure were significant: Rehearsal × Similarity, *F*(1, 15) = 26.64, *p* < .001 (*η*_*p*_^*2*^= .64); Rehearsal × Serial Position, *F*(5, 75) = 5.79, *p* = .002 (*η*_*p*_^*2*^ = .28), Greenhouse–Geisser correction applied. The interaction of similarity structure and serial position was also significant, *F*(1, 15) = 31.35, *p* < .001 (*η*_*p*_^*2*^ = .68). Although this interaction has no direct bearing on our predictions, its consistency with previous reports should be noted. Finally, the three-way Rehearsal Strategy × Similarity Structure × Serial Position interaction was not significant, *F*(5, 75) = 2.17, *p* = .091 (*η*_*p*_^*2*^ = .13), Greenhouse–Geisser correction applied.

The significance of both two-way interactions with rehearsal strategy provides preliminary evidence that serial recall performance for alternating lists of similar and dissimilar items is subject to the influence of emergent sequential properties. These are not reducible to properties of the individual items, since the sequences themselves are, item by item, identical, and across grouping conditions they still maintain the same within-list similarity structure.

Examination of Fig. 3 suggests several other performance characteristics that are problematic to a purely item-level account: notably, the apparently less scalloped (and lower) performance curve for DS sequences under pairs than under triplets grouping, and the equivalent performance levels for similar and dissimilar items in several instances under both rehearsal conditions. For example, triplets rehearsal of SD sequences gives rise to similar performance at Serial Positions 3 and 4, whilst pairs rehearsal of DS sequences induces similar performance at Serial Positions 2 and 3. The equivalence in performance is particularly striking when comparing recall of the S items at initial and terminal sequence positions to that of their adjacent D items. In three out of four cases (triplets SD-initial, triplets DS-terminal, and pairs DS-terminal), no cost of similarity is apparent, whilst in the case of SD-initial pairs, the slope of the first saw-tooth appears shallow relative to those in the remaining plot.

In order to confirm these effects, we first performed separate repeated measures ANOVAs within each rehearsal condition (pairs, triplets), and then used planned contrasts within each combination of similarity and rehearsal (SD pairs, SD triplets, DS pairs, and DS triplets) to establish the detailed pattern of task performance.

#### Pairs

Under pairs rehearsal, both main effects were significant: similarity structure (SD > DS), *F*(1, 15) = 20.31, *p* < .001 (*η*_*p*_^*2*^ = .58), and serial position, *F*(5, 75) = 16.25, *p* < .001 (*η*_*p*_^*2*^ = .52). The two-way interaction was also significant, *F*(5, 75) = 19.96, *p* < .001 (*η*_*p*_^*2*^ = .57). In order to quantify the strength of each saw tooth, SD and DS sequences were then analyzed separately, using a one-way repeated measures ANOVA with planned (repeated) contrasts (see Table 1). In SD sequences, all contrasts were significant, confirming the subjective appearance of the saw tooth. However, in DS sequences, only two contrasts reached significance: the comparison between Serial Positions 1 and 2, and the comparison between Serial Positions 3 and 4—[+1, –1, 0, 0, 0, 0], *F*(1, 15) = 28.5, *p* < .001, and [0, 0, +1, –1, 0, 0], *F*(1, 15) = 9.76, *p* = .007.

**Table 1:** Experiment 1: *F* values and the accompanying significances of repeated contrast metrics for SD and DS sequences under pairs and triplets grouping/rehearsal conditions

	Pairs				Triplets			
	SD		DS		SD		DS	
Contrast	*F*(1, 15)	*p*	*F*(1, 15)	*p*	*F*(1, 15)	*p*	*F*(1, 15)	*p*
[+1, 0, 0, 0, 0]	10.4	.006	28.5	<.001	0.05	.825	14.1	.002
[0, +1, 0, 0, 0]	59.7	<.001	0.132	.722	19.5	<.001	8.01	.013
[0, 0, +1, 0, 0]	34.3	<.001	9.76	.007	2.62	.126	49.8	<.001
[0, 0, 0, +1, 0]	27.1	<.001	1.69	.213	8.75	.010	10.6	.005
[0, 0, 0, 0, +1]	27.3	<.001	34.2	.706	24.7	<.001	0.267	.267

#### Triplets

Under triplets rehearsal, the main effect of serial position only was significant, *F*(1, 15) = 16.02, *p* < .001 (*η*_⁯_^⁯^_p_^2^ = .52). The Similarity × Serial Position interaction was also significant, *F*(5, 75) = 20.50, *p* < .001 (*η*_p_^2^_⁯_^⁯^ = .57). Contrast metrics in the SD sequences revealed significant differences at Serial Positions 2 versus 3, 4 versus 5, and 5 versus 6. In DS sequences, all contrast metrics were significant, with the exception of Items 5 versus 6. A summary of all the *F* values and their accompanying significance probabilities is presented in Table 1.

#### Transposition errors

**Fig. 4:**
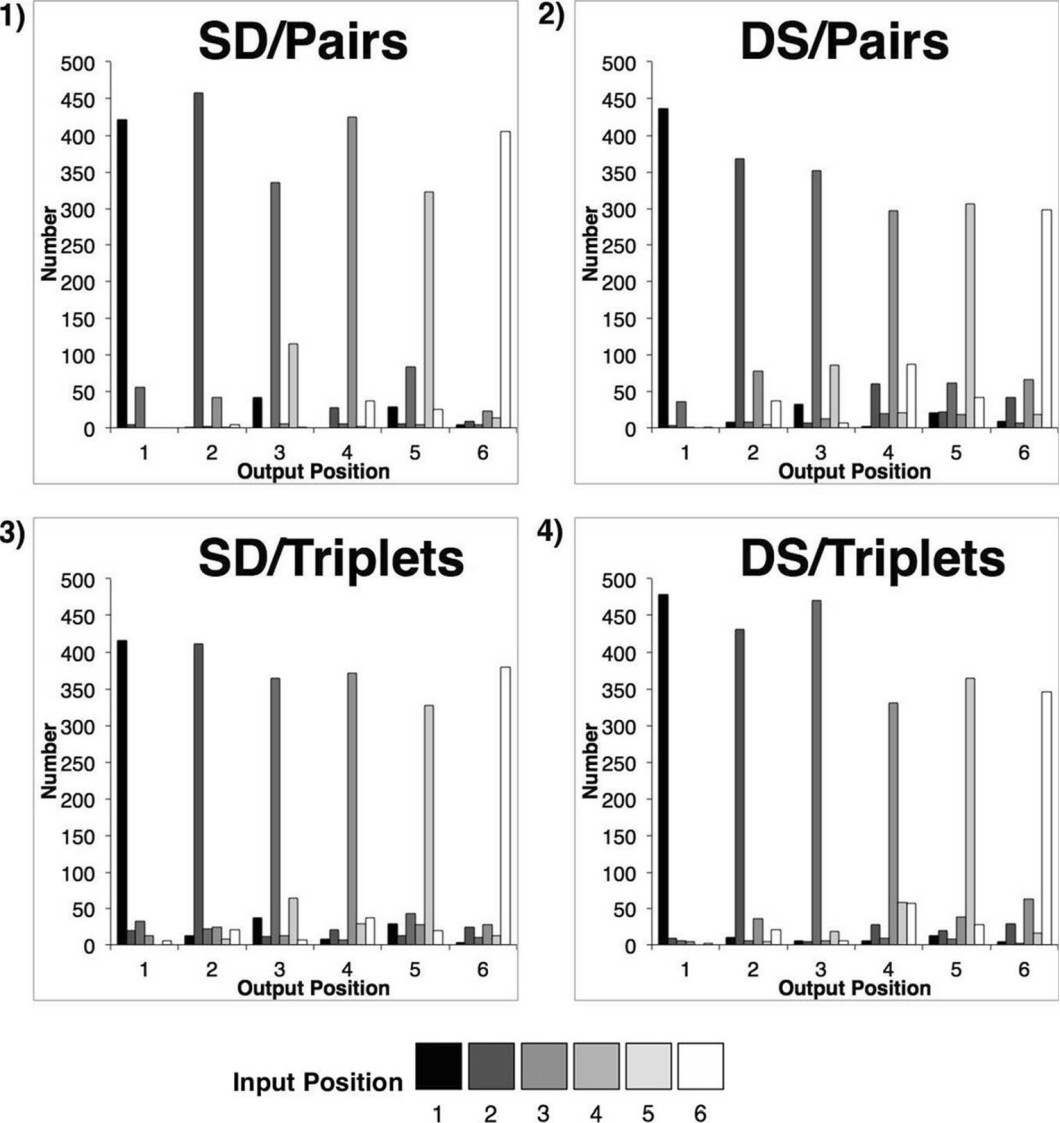
Transposition gradients showing total numbers of items recalled at each input–output pairing under pairs (panels 1 and 2) and triplets (panels 3 and 4) grouping. The input positions (denoted by shading) are arranged left to right at each output position

**Fig. 5:**
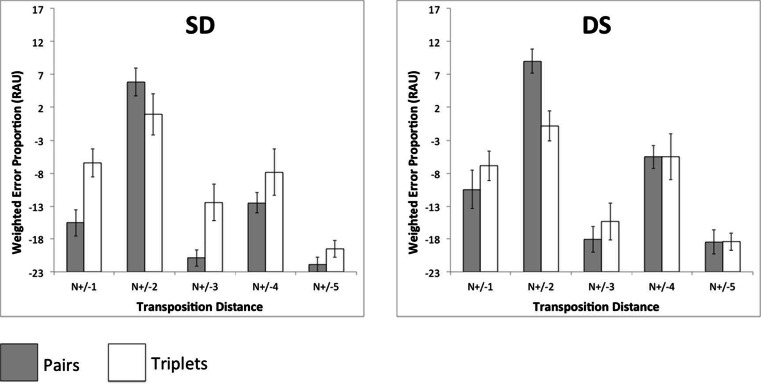
Weighted proportions of transposition errors at five successive transposition distances, obtained under pairs and triplets rehearsal, for alternating heterogeneous sequences. Values are expressed as rationalized arcsine units. Error bars denote standard errors

The total number of transposition errors was calculated for each pair of input–output positions in each condition (Fig. 4). These data were then collapsed across output positions, allowing errors to be plotted as a function of transposition distance (Fig. 5). Errors were then expressed as weighted proportion scores—namely, the number of transpositions at each distance, expressed as a proportion of the total number of possible transpositions at that distance. Since an intrusion or omission at a given serial position rendered a transposition to or from that position impossible, the total number of possible transpositions was adjusted on a case-by-case basis to exclude these errors. It should be noted, however, that these errors were rare and had no appreciable impact on the values reported.

In the analysis that follows, we have focused on a restricted range of transpositions—up to a distance of *N*_(±2)_. The reasons for this are twofold. The first relates directly to our hypothesis: We predicted that any effect of grouping and rehearsal would become clearly manifest in modulations at distances of *N*_(±1)_ and *N*_(±2)_. Thus, failure to detect significant error modulation at distances up to *N*_(±2)_ would itself be grounds to reject our hypothesis. The second is a matter of statistical robustness: The locality constraint (Henson et al., 1996) predicts that the largest number of transposition instances will occur at short input–output distances.

In order to compare the mean transposition likelihoods at different input–output distances, the raw error counts were converted to weighted log odds (cf. Henson et al., 1996). This transformation takes account of range effects in proportion data, but additionally corrects for differences in variances associated with means being calculated from differing population sizes.

We first tested the hypothesis that the two rehearsal conditions had differential impacts on transposition errors. Weighted log-odds error scores for the collapsed (SD and DS) similarity structures were subjected to a 2 (rehearsal: pairs, triplets) × 2 (direction: plus, minus) × 2 (serial distance: *N*_(2)_, *N*_(1)_) repeated measured ANOVA. None of the two- and three-way interactions with transposition direction were significant (*p*s > .05). The data were therefore collapsed across directions and subjected to a 2 (rehearsal: pairs, triplets) × 2 (serial distance: *N*_(±2)_, *N*_(±1)_) repeated measured ANOVA. The main effect of serial distance was significant, *F*(3, 45) = 20.2, *p* < .001, whilst the main effect of rehearsal was not (*F* < 1). Crucially, the two-way interaction was significant, *F*(3, 45) = 32.6, *p* < .001, confirming the pattern shown by Fig. 3, that rehearsal in pairs promotes transpositions between parallel sequence locations, both by increasing the error likelihoods at *N*_(±2)_ and decreasing the error likelihoods at *N*_(±1)_. Direct comparisons between the weighted log-odds for pairs and triplets at each transposition distance supported this conclusion: *N*_(–2)_, *Z* = 7.80, *p* < .001; *N*_(–1)_, *Z* = –4.06, *p* < .001; *N*_(+1)_, *Z* = –3.14, *p* = .002; *N*_(+2)_, *Z* = 5.46, *p* < .001. In other words, *Z* values were significantly positive for *N*_(±2)_ transpositions and significantly negative for *N*_(±1)_ transpositions.

Since our predictions speak directly to the effect of rehearsal on the transposition of similar items, errors were analyzed separately for S items in each grouping condition. Direct comparisons between the weighted log-odds for pairs and triplets at each transposition distance confirmed the effect of rehearsal on error likelihoods for S items and that the effect specifically reflected modulation at *N*_(±2)_: *N*_(–2)_, *Z* = 2.76, *p* = .006; *N*_(+2)_, *Z* = 3.20, *p* = .001; *N*_(–1)_, *Z* = 0.68, *p* = .50; *N*_(+1)_, *Z* = 0.37, *p* = .71. The rehearsal manipulation can thus be seen to directly influence the likelihood of S-item parallel transpositions, such that errors of this type are more likely to occur when material is rehearsed in pairs rather than triplets. However, analysis of the D-item errors identified a transposition pattern highly similar to that found with S items: *N*_(–2)_, *Z* = 2.51, *p* = .012; *N*_(+2)_, *Z* = 3.08, *p* = .002; *N*_(–1)_, *Z* = 0.68, *p* = .49; *N*_(+1)_, *Z* = –0.53, *p* = .59. Taken together, the S and D transposition analyses imply that item-wise similarity—although reflected somewhat in the serial position curves—either provides an incomplete account of transposition likelihood or is itself susceptible to modification by emergent stress metrics.

Having established a general principle that the pattern of transposition in serial recall is both somewhat malleable and task-dependent rather than solely determined by item-level characteristics, we turned to a related question: Given the asymmetry of the SD and DS serial position graphs under both rehearsal schemes, do these asymmetries have a basis in qualitatively different error patterns? To address this question, the weighted log-odds were subjected to a 2 (rehearsal: pairs, triplets) × 2 (structure: SD, DS) × 2 (distance: *N*_(±1)_, *N*_(±2)_) repeated measures ANOVA. The critical Grouping × Structure interaction was significant, *F*(1, 15) = 4.64, *p* = .048, revealing that error patterns are indeed dependent on both rehearsal and similarity structure (Fig. 5). In terms of our hypothesis, this suggests strongly that the emergence of divergent stress patterns during rehearsal is not only an important (and previously neglected) determinant of recall performance, but that the stress patterns themselves may be subtly altered by the detailed syllabic structure of the targets.

### Discussion

In Experiment 1, we demonstrated that during serial recall of alternating lists of similar and dissimilar items, performance levels (as revealed by the serial position curves) and transposition patterns could be modulated simply by guiding articulatory grouping via an explicit rehearsal strategy. As we anticipated, the rehearsal manipulation led to divergent prosodic structures in the to-be recalled sequences, with paired and triplet strategies inducing trochaic and cretic stress meters, respectively, albeit with the latter rendered more reliably than the former.

The emergence of divergent stress distributions under the different rehearsal conditions is interesting in two respects. First, it illustrates the emergence of a series of uniform properties that might serve to impose constraints on how a sequence is articulated during rehearsal. For example, since spoken English words are predominantly stress-initial, stressed sequence locations may serve to demarcate sequences into smaller articulatory events (i.e., objects) with word-like properties. Second, the more robust production of the cretic stress pattern, as compared to the trochaic, suggests that the reliabilities and stabilities of articulatory control and execution are unequal for the two rehearsal conditions.

Quantitatively, performance approximated the saw-tooth profile widely reported in other studies. Although the saw-tooth performance in alternating sequences could derive from item-based similarity, it is clear that the DS and SD sequence orders were neither symmetrical within, nor equivalent between, rehearsal conditions. Examination of the overall error patterns revealed up-modulation of *N*_(±2)_ errors coupled with down-modulation of *N*_(±1)_ errors under pairs (vs. triplets) rehearsal. Furthermore, transpositions differed within the SD and DS sequences. Specifically, the down-modulation of errors at *N*_(±1)_ was restricted to SD sequences, whilst up-modulation of *N*_(±2)_ errors occurred in both sequence types. In other words, no simple Item × Stress relationship can adequately capture the shape of the data. It is important to note, however, that *within* each rehearsal condition, the pattern of errors does closely resemble those reported by Henson et al. (1996), and the comparison between rehearsal strategies is what reveals the sequence-level effects.

Although item-wise similarity is widely identified as the basis of the saw-tooth performance profile in serial recall of alternating sequences, our data suggest that considering similarity as the sole determinant of recall likelihood is mistaken. Instead, we propose that it is more useful to envisage the stimulus sets employed in serial recall tasks in terms of the properties of the entire sequences that emerge from their intended use. As we suggested previously, in the case of sequences derived from closed sets of meaningless, novel consonants, such as those employed in the present experiment, item–item similarity may well exert an influence. However, even for such structurally impoverished sequences, the instruction to rehearse in pairs and triplets will act to guide the formation of articulatory objects, whose structures are defined by the onset and offset of the grouping. In other words, even when the sequences afford an item-based analysis, structured rehearsal defines a series of prosodic relationships that may interact differently with syllabic structure, with further dependency on the stability of the prosodic features. For example, in pairs rehearsal, alternating stress patterns in the alternating lists will phase-align, both with each object boundary and with onset–rime features within each successive object (e.g., D-s, D-s, D-s). This contrasts with triplet grouping, where stress is reliably asymmetric (e.g., cretic), and thus out of phase with the similarity structure (e.g., D-s-D, S-d-S). This may, for example, act to stabilize the articulatory object in the triplet grouping (relative to the more weakly defined trochaic pair) by engendering greater and more stable articulatory contrast within the sequence as a whole.

Although we have argued that the asymmetric saw-tooth patterns and divergent transposition patterns reported above derive from divergences in the articulatory control process, other factors cannot be excluded. For instance, under both grouping conditions, the *relative* likelihood of an error at a given output position conforms to the constraints of locality and similarity reported elsewhere (e.g., Henson et al., 1996), and the overall pattern of errors is congruent with the alternating pattern of similar and dissimilar items. An alternative explanation could therefore be that although articulatory control at the sequence level has a modulatory effect on item recall, the gross pattern of errors still derives from the similarity structure of the sequences rather than from the articulatory plan per se*.* However, another way to interpret this relationship would be that the patterning of phonological structure across these alternating sequences induces participants more readily (and even, to some degree, against explicit instructions otherwise) into embodying the sequence in a particular prosodic form—namely, a trio of pairs—and that this is the main driver of the particular pattern of performance, rather than the properties of the items within that structure themselves.

In Experiment 2, we examined this possibility by testing serial recall performance for homogeneous lists under the same conditions employed in Experiment 1. Since homogeneous sequences (by definition) lack alternating patterns of item similarity, if the manipulation of rehearsal were simply acting to modulate preexisting error patterns originating in the item-level similarity structures, then we would expect the transposition errors in homogeneous sequences to be immune to the influence of stress. Instead, transposition gradients should be shaped by locality constraints (i.e., Henson et al., 1996), irrespective of rehearsal. Conversely, if grouping and rehearsal could be shown to elicit a crossover in error patterns in the absence of alternating similarity, then such item-similarity relationships could be eliminated as being, necessarily, the causal agent responsible for the transposition error patterns reported in Experiment 1.

## Experiment 2

### Method

#### Participants

A group of 16 participants (12 female, four male; mean age 20 years) were recruited from the Cardiff University Human Participant Panel. Informed consent was obtained in accordance with Cardiff University School of Psychology ethics procedures.

#### Materials

The stimuli comprised the two homogeneous sets (S and D) described in Experiment 1. Thirty-six permutations of each set were obtained using the same slot-by-slot item transpositions described in Experiment 1.

#### Design and procedure

A 2 (grouping structure: pairs, triplets) × 2 (similarity: S, D), within-subjects design was employed. Each participant performed two experimental runs (one of each grouping structure). Each run comprised four blocks (two each of S and D). Each block comprised 16 sequence permutations sampled randomly (without replacement) from each list. Block orders were constructed with the constraint that each half of each run comprised one S and one D block. This gave a total of eight block orders crossed with two run orders, and each permutation was tested once. The testing procedures were identical to those of Experiment 1.

### Results

#### Stress

**Fig. 6:**
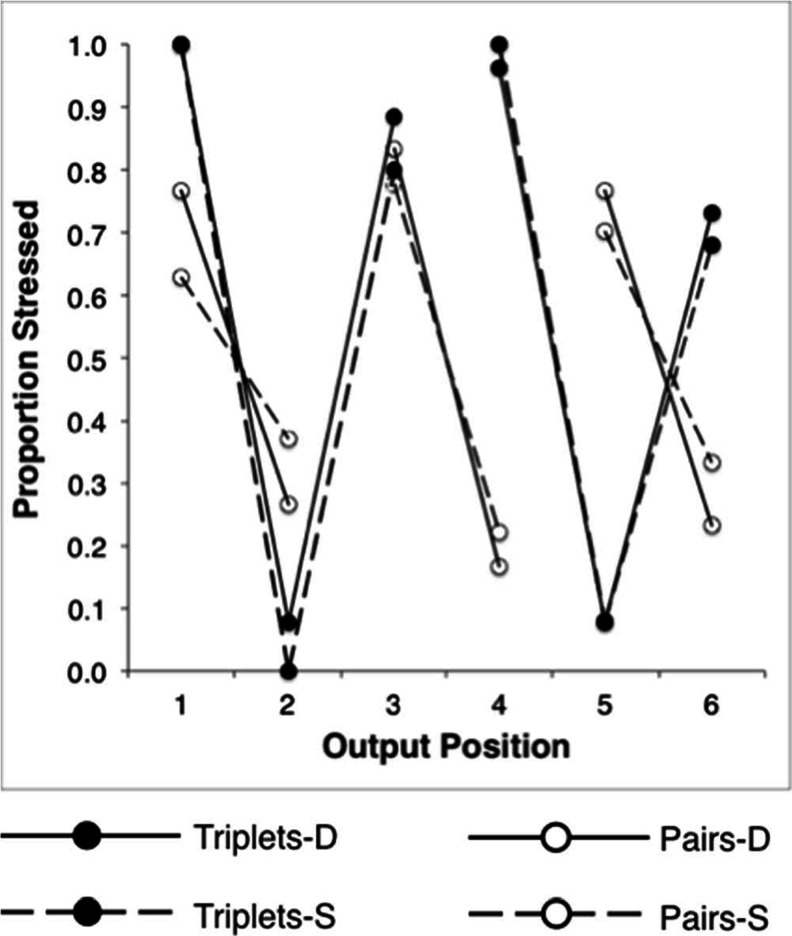
Prosodic stress probabilities for six-item homogeneous sequences, conforming to D (solid lines) and S (broken lines) structures, during recall under pairs (open circles) and triplets (closed circles) grouping

Stress patterns were characterized using the method described in Experiment 1. Once again, the two groupings gave rise to distinct stress patterns (Fig. 6) in both correctly and incorrectly recalled sequences. The test–retest stress attributions were again highly correlated, *r*(24) = .91, *p* < .001 (two-tailed), indicating reliable categorization.

#### Serial position curves

**Fig. 7:**
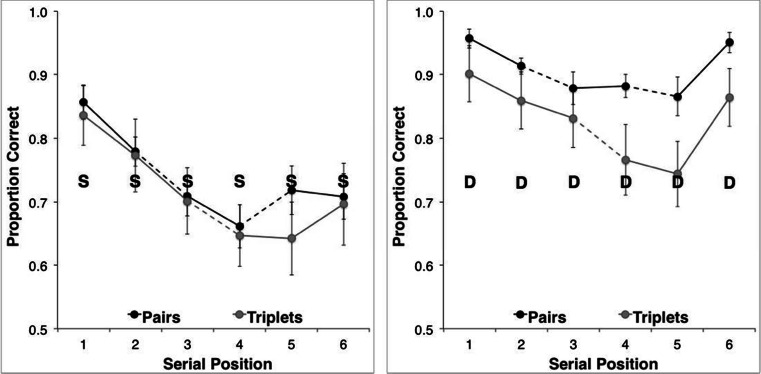
Serial position plots depicting mean (*N* = 16) performance obtained for homogeneous similar (S, left panel) and dissimilar (D, right panel) six-item sequences, under two rehearsal strategies. Error bars denote standard errors

These data are presented graphically in Fig. 7. Serial position data were subjected to a 2 (rehearsal: pairs, triplets) × 2 (similarity: D, S) × 6 (serial position) repeated measures ANOVA. To counter the potential floor and ceiling effects inherent in proportion data, the raw proportion scores were subjected to an arcsine transformation prior to statistical analysis. The main effects of both similarity and serial position were significant: *F*(1, 15) = 43.3, *p* < .001, and *F*(5, 75) = 28.5, *p* < .001, respectively. The remaining interactions were not significant. Quantitatively, then, the data suggest no effect of organization via rehearsal in the absence of an alternating similarity structure, as well as a general decline in recall from primacy to midlist.

#### Transposition errors

**Fig. 8:**
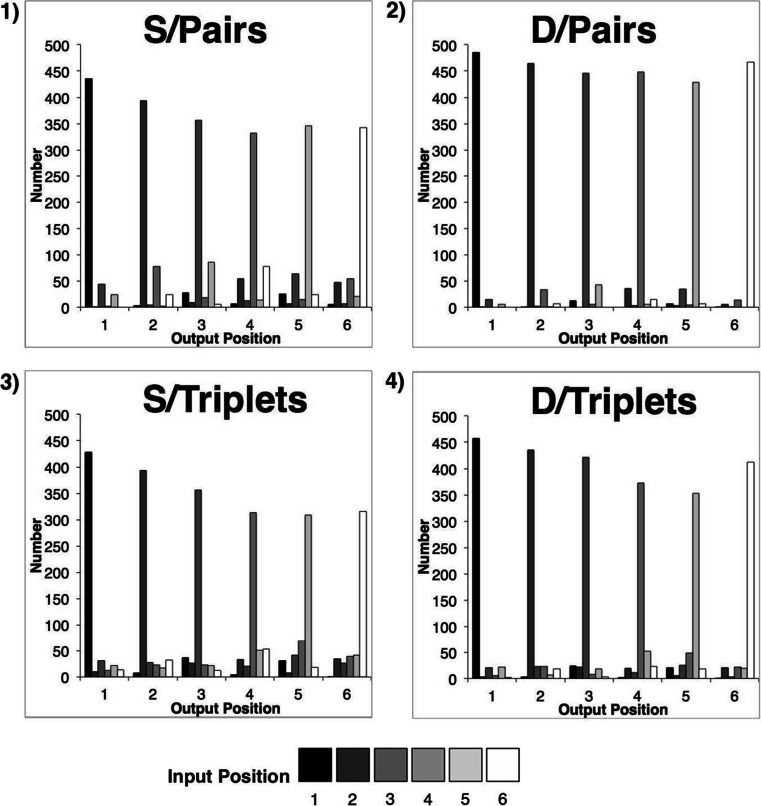
Transposition gradients showing total numbers of similar (panels 1 and 3) and dissimilar (panels 2 and 4) items recalled at each input–output pairing under pairs (panels 1 and 2) and triplets (panels 3 and 4) rehearsal. The input positions (denoted by shading) are arranged left to right at each output position

Transposition gradients for the S and D sequences are presented in Fig. 8. When considering the patterns of errors found in homogeneous lists, it should be noted that the error count in D sequences is ~50 % of that found in S lists. Given the risk of floor effects, the D error data will only be considered qualitatively. Accepting this caveat, it is clear that in terms of transposition distances—particularly the dominant *N*_(±1)_ and *N*_(±2)_—S and D items behave qualitatively very similarly to each other under both grouping strategies (Fig. 8). Both the similar and dissimilar sequences exhibit an alternating transposition pattern under pairs rehearsal in which nonadjacent errors are dominant—a pattern that, critically, was obtained in the absence of any alternating similarity structure. This was not the case in the triplets rehearsal condition.

Thus, the transposition gradients obtained in Experiment 2, using homogeneous sequences, are consistent with those reported for alternating sequences in Experiment 1 (Fig. 4). This finding supports our hypothesis that the modulatory effects of rehearsal on transposition likelihoods are not dependent in the first instance on item-wise similarity. The prevalence of *N*_(±2)_ errors in the absence of a saw-tooth serial position curve (see Fig. 7) implies that *all* items in the S sequences under pairs rehearsal must be equally likely to transpose to *N*_(±2)_. The transposition gradients for S sequences confirm that this was indeed the case (Fig. 8, panel 1), with alternating (*N*_(±2)_) errors dominating all nonunity output positions. This pattern contrasts sharply with the same sequences recalled under a triplets rehearsal regime. Here (Fig. 8, panel 3), *N*_(±1)_ transpositions are more distributed, with nearest-neighbor transpositions usually being as likely as alternating transpositions.

**Fig. 9:**
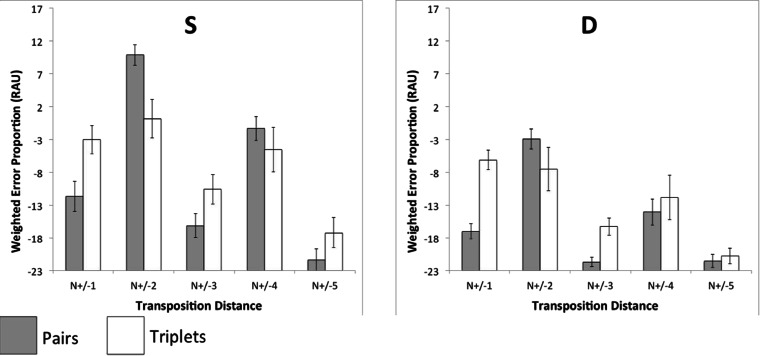
Weighted proportions of transposition errors at five successive transposition distances, obtained under pairs and triplets rehearsal, for homogeneous similar (S) and dissimilar (D) sequences. Values are expressed as rationalized arcsine units. Error bars denote standard errors

These impressions were confirmed statistically. Transposition errors were collapsed across input–output positions, allowing for the comparison of errors rates as a function of transposition distance (Fig. 9).

In order to establish whether the transposition patterns were equivalent in both positive and negative directions, the weighted log-odds of error proportions were subjected to a 2 (rehearsal: pairs, triplets) × 2 (direction: plus, minus) × 2 (transposition distance) repeated measures ANOVA. All interactions with transposition direction were nonsignificant (*F*s < 1 in all cases). In order to test the hypothesis that pairs rehearsal led to up-modulation of *N*_(±2)_ transpositions and down-modulation of *N*_(±1)_ transpositions, relative to triplets rehearsal, the data were then collapsed across transposition directions and subjected to a 2 (grouping: pairs, triplets) × 2 (transposition distance) repeated measures ANOVA. The main effect of rehearsal was not significant (*p* = .72), but the main effect of transposition distance was significant, *F*(1, 15) = 10.0, *p* = .006, and the critical two-way Transposition Distance × Grouping interaction was also significant, *F*(1, 15) = 30.7, *p* < .001. These data confirm that the malleability of transposition error patterns reported for alternating lists in Experiment 1 was maintained for homogeneous sequences. Specifically, rehearsal in pairs (relative to rehearsal in triplets) acts to increase the likelihood of transpositions occurring between alternating sequence locations and to decrease the likelihood of transpositions occurring between adjacent sequence locations, irrespective of item–item similarity.

### Discussion

In Experiment 2, transposition errors in homogeneous sequences were modulated qualitatively by rehearsal condition. Under pairs rehearsal, alternating, nonadjacent (i.e., *N*_(±2)_) errors were predominant for both S and D sequences, closely mimicking the performance obtained here and elsewhere for alternating similar/dissimilar sequences. Critically, the error pattern was obtained in the absence of a saw-tooth serial position curve, with alternation being equally likely at all output positions. This argues strongly against the idea that alternation of phonological similarity within a list is a necessary determinant of alternating errors, and further demonstrates that alternating errors are themselves not sufficient to determine saw-tooth performance.

This apparent decoupling of the relationship between the patterns of item similarity, transposition likelihood, and saw-tooth recall performance provides new evidence that ostensible item-level stimulus properties cannot fully account for the patterns of errors found in serial recall. Triplets rehearsal induces a different error pattern, largely dominated by locality. Given that the sequences were identical, item by item, in the two conditions, differing only in their spatial arrangements at presentation and their prosodic structures during rehearsal and recall, we are led to conclude that the sequence-level properties arising from the differential task requirements in the two conditions interact significantly with item-level stimulus properties to determine errors in serial recall.

## General discussion

In two experiments, we demonstrated that the mechanism underlying transposition errors in serial recall is subject to significant influence from prosodic factors such as stress. Experiment 1 showed that for alternating sequences of similar and dissimilar items, a simple rehearsal manipulation modulated both the shape of the classical saw-tooth serial position curves and the likelihood of transposition between pairs of input–output positions. Relative to triplets, pairs rehearsal increased the likelihood of transposition at output distances *N*_(±2)_ from input and attenuated the transposition likelihood at *N*_(±1)_. By way of contrast, the pattern of errors that emerged following rehearsal in triplets was predominantly characterized by *N*_(±1)_ transpositions, despite the item-level alternating similar/dissimilar structure. In Experiment 2, the modulating effect of rehearsal on transposition error likelihoods was replicated for homogeneous sequences. For both S and D sequences, rehearsal in pairs led to a predominance of *N*_(±2)_ transpositions, whereas the transpositions arising from rehearsal in triplets were more likely to be *N*_(±1)_. This demonstrates that the influence of rehearsal on transposition error patterns is not dependent on the presence of an alternating pattern of item similarity. However, the divergent patterns of errors were not reflected in the shapes of the serial position curves, which conformed to a broadly U-shaped profile, irrespective of whether pairs or triplets rehearsal was employed.

Attempting to account for this pattern of results within existing theoretical frameworks raises several important issues: Firstly, we need to consider the likely locus of the effect of the stress manipulation. Many accounts of vSTM have proposed that serial order is encoded and retrieved by means of some form of order representation that is independent of the phonological properties of the to-be-recalled items. Such approaches typically posit that phonology comes into play to the extent that it influences competitive cueing at retrieval. Influential examples of this type of order-encoding system include primacy gradients (e.g., Grossberg, 1978; Page & Norris, 1998), start–end markers (e.g., Henson, 1998), and context/timing signals (e.g., Brown et al., 2000; Burgess & Hitch, 1999; Hitch, Flude, & Burgess, 2009). If metrical stress simply influences the encoding/retrieval context of the item order, then the present results may potentially be understood as the outcome of perceptual-grouping phenomena, rather than as any direct modulation of phonological similarity.

Accounting for the present data in terms of grouping phenomena, then, is contingent on the translation of the written structure of a simultaneous visual presentation into a temporally grouped context signal, and we suggest that the most plausible mechanism by which this could occur is that of subvocal articulation during reading, and more especially during rehearsal. We therefore reiterate Wickelgren’s (1964) assertion: “Whatever else a grouping method is, it is a method of rehearsal” (p. 414). Interestingly, in earlier work exploring the effects of grouping on serial recall using the same conceptual framework (albeit in a less fully developed form), Hitch, Burgess, Towse, and Culpin (1996) provided evidence for an articulatory influence on grouping. Local recency effects in visual (but not auditory) lists were attenuated (although not entirely abolished) by articulatory suppression, and this effect was most evident in early list positions, where list items are most likely to enter a rehearsal cohort. Converging evidence for rehearsal-based grouping can also be seen in Frankish (1985), where triplets rehearsal of visually grouped and ungrouped sequences gave rise to qualitatively similar patterns of local recency. Furthermore, the emergence of scalloping in serially presented, visually grouped lists has been shown to depend on intergroup timing—and thus on the opportunity to engage in rehearsal (Frankish, 1989).

Modulation of recall performance by various types of grouping manipulation is not in itself a new finding (Burgess & Hitch, 2006; Frick, 1989; Henson et al., 1996; Hitch et al., 1996; Hitch et al., 2009; Reeves et al., 2000; Ryan, 1969), and although the proposed mechanisms vary between models, the broad consensus is that grouping gives rise to hierarchically organized positional cues, separately coding the positions of items within each group and within each sequence (cf. Wickelgren, 1964, 1967). For example, in their neural-network model of the phonological loop, Burgess and Hitch (2006) presented simulations of grouping in serial recall for both auditory and visual lists, which demonstrated a close fit to empirical data. The model accounts for grouping by explicitly tracking within-group timings in addition to within-list timings. The effects of grouping arise due to the increased temporal distinctiveness of end-of-group items relative to midgroup items. Similarly, in the start–end model of Henson (1998), separate start–end markers are proposed for both the group and the overall sequence, allowing for the encoding of two dimensions of order. Irrespective of the underlying mechanism, an important consequence of hierarchical grouping is its effect on transposition error patterns: Transpositions *between* groups are predicted to maintain their positional integrity *within* groups (Henson, 1998).

Thus, for the six-item lists employed here, we should expect to see significant differences in the relative proportions of errors for the two rehearsal conditions at distances of *N*_(±2)_ and *N*_(±3)_, since these would represent two- and three-item group boundaries, respectively. However, examination of Figs. 8 and 9 reveals a somewhat different pattern of results. Whilst the effects of a pair-wise rehearsal strategy does predominantly induce *N*_(±2)_ transpositions, consistent with the preservation of within-group position, triplets rehearsal primarily increases *N*_(±1)_ transpositions, whilst exerting only a minor influence at *N*_(±3)_. Indeed, the overall pattern of transpositions shown in Fig. 9 is difficult to reconcile with an account based on hierarchical grouping. This is not to suggest that models of grouping phenomena are themselves incorrect, but rather to suggest that grouping itself does not reliably account for the results reported here.

A commonly reported feature of grouped recall generally is the within-sequence local recency effect: a decline in error rates for the final item in each recalled group, reflecting the particular grouping strategy employed (Madigan, 1980). Such recency has been obtained both with auditory and visual presentation (Frankish, 1985). Furthermore, many studies have claimed a general advantage for triplets grouping (see also Henson et al., 1996) over ungrouped and all other groupings, originating either in chunking constraints (Cowan, 2010; Ryan, 1969) or phonological storage constraints (Hitch et al., 1996; but see Frankish, 1985, 1989).

The present data cast some doubt both on the generality of the reported effects and on their likely mechanism, on two grounds. First, in our experiments, serial position curves reveal no quantitative advantage for triplet over pairs rehearsal in either homogeneous or alternating-sequence recall. Second, local recency is not a consistent feature of the data reported here. This is particularly striking in Experiment 2, in which the homogeneous lists provided the clearest opportunity for local recency effects to emerge. In the absence of an alternating similarity structure, a steady decline in recall performance was predicted at successive list positions; in other words, a smooth curve with no scalloping should have emerged when lists were homogeneous. If they were present, local recency effects should therefore have been easily detectable, since they would become manifest as scalloping in the serial position curve. If the effects of stress were not readily describable in terms of the influence of hierarchical grouping on serial order, it might be argued instead that stress patterns emerging during rehearsal simply acted to modulate preexisting item-level patterns of similarity, and that the transposition gradients therefore still reflect essentially the alternating similar/dissimilar structure. On this question, the data are less conclusive. If it were the case that an alternating pattern of similarity is a necessary prerequisite for the influence of stress on transposition likelihood, then divergent stress patterns would not be expected to have the same effect on transposition gradients for homogeneous as they do for alternating sequences. However, in Experiment 2, the qualitatively distinct modulations of the transposition gradients under the two rehearsal strategies were replicated for homogeneous sequences. This indicates that an item-level, alternating similarity structure is not a prerequisite for such a pattern. However, the emergence of divergent prosodic structures during rehearsal did not give rise to complementary differences in the shapes of the serial position curves, as might be predicted if sequence-level properties such as metrical stress were the sole determinant of recall errors.

Indeed, differences between the curves were slight and were restricted to a single serial position for both S and D sequences. Although this pattern of results could be interpreted as support for a mere modulatory account of rehearsal, the finding is in fact somewhat paradoxical. It is widely assumed in item-based accounts (following Henson et al., 1996) that there is a causal link between absolute transposition likelihoods—based on the dual constraints of locality and similarity—and the shape of the serial position curve. Any process that differentially modulates transposition error likelihoods should therefore also give rise to deviations in the serial position curves. This was empirically not the case in Experiment 2. Since the transposition likelihoods for S items were of similar magnitudes in Experiments 1 and 2, the absence of a saw-tooth character in the latter experiment cannot simply be attributed to quantitative differences in the susceptibility of the homogeneous sequences to the rehearsal manipulation. We therefore conclude that although the results of Experiment 2 do demonstrate the influence of articulatory processes on serial recall that represent more than the modulation of preexisting item-based effects, prosodic factors alone appear insufficient to fully account for all aspects of recall performance.

Our emphasis on the role of articulatory processes in defining the pattern of transposition errors differs from the prevailing approaches to grouping effects adopted by computational models of serial recall, in which sources of error have typically been ascribed to retrieval constraints, based on item distinctiveness. One possible resolution would be to suggest a more “relaxed” item-based account, in which the representational format of the to-be-remembered material would still be fundamentally reliant on phonological features, but in addition, emergent sequential properties (e.g., stress markers) would give rise to auxiliary item features, which although not strictly phonological in nature, would modify the similarity between any two competing items at retrieval. In other words, we may simply need to adopt a more liberal definition of the concept of “the item,” such that some item features are immutable and others (such as stress markers) arise ad hoc. Such a proposal might be seen as broadly compatible with the feature model of immediate memory (Nairne, 1990), if stress markers can be seen to act as modality-dependent features in primary memory. However, some caveats do apply here. Although “items” can be classified as being either stressed or unstressed, that difference is relational and not reducible to any single property of the item. Secondly, the degree to which stress is modality-dependent is open to question, since despite stress being an auditory property, the present data indicate that it can also emerge during the subvocal rehearsal of visually presented stimuli. Despite this, it is still possible that the unified perceptual property that is experienced as “stress” may be marked by more than one representational feature. Indeed, some evidence does support such a proposal. Maybery, Parmentier, and Jones (2002) showed that in a serial recall task, the timing of grouping effects during recall were independent of the ratio of within-group to between-group pauses at input. This illustrates that the input properties of the sequence might vary considerably, yet give rise to a consistent prosodic marker (i.e., timing) at output.

There is precedent for the degree of malleability that we found in the transposition likelihoods within serially recalled sequences. The likelihood of correct recall at a given sequence position has been shown to depend both on the ratio of similar- to dissimilar-sounding items in the sequence and on the range of possible alternative items from which a participant might make a selection (Farrell & Lewandowsky, 2003). Accordingly, a dissimilar item contained within a sequence that otherwise comprises similar-sounding items is recalled better than when it is located within a sequence of mutually dissimilar items (the isolation effect). A recall advantage is also found for dissimilar items in alternating (similar, dissimilar) sequences when compared to recall from mutually dissimilar sequences. Correspondingly, increasing the range of available (incorrect but plausible) responses has been shown to decrease the likelihood of correct recall (e.g., Farrell, 2006; Farrell & Lewandowsky, 2003; Lewandowsky & Farrell, 2008). For example, if the total number of items employed across all trials in a recall experiment (i.e., the ensemble) is greater than the number of items deployed in each trial, errors are liable to increase in the form of incorrect recall of nontrial ensemble items. By the same token, intrusion errors can be minimized if test items are re-presented for order reconstruction rather than demanding overt recall (Farrell & Lewandowsky, 2003). However, we consider it unlikely that the error patterns reported in the present data are attributable to these factors, since in the experiments reported here ensemble size was explicitly controlled, participants were allowed to familiarize themselves with each ensemble, and both intrusion and omission errors were so rare in our data as to have no significant effect on the shapes of the serial position curves. Since mechanisms related to guessing strategies could be eliminated—in addition to those arising from perceptual grouping—we suggest that the reported transposition gradients reflect processes engaged by the rehearsal demands of the task.

More generally, and on the basis that the item-by-item sequence contents were equivalent in the two rehearsal conditions, we propose that explanations focusing primarily on item-level similarity offer an inadequate account of the data and that task performance can only be accounted for by considering these alongside the role of sequence-level properties that are not reducible to features of the constituent items, but that instead derive from articulatory constraints that emerge during subvocal rehearsal of the whole sequence, interacting dynamically with the actual content of that sequence. This is not to say that item-level descriptions make no contribution to task performance. Empirically, in Experiment 2, the dominance of *N*_(±2)_ transposition errors in the absence of an accompanying saw-tooth serial curve profile indicates that emergent sequence properties offer an incomplete account of recall performance. Indeed, in the paradigm employed here (and commonly in serial recall experiments), the meaningless concatenations of consonants that comprised the to-be-remembered material differed markedly from the content of natural speech and clearly allowed an item-based analysis. One possible resolution might be to reconsider the dichotomy between the properties of a priori items versus emergent sequences. Whereas coarticulation guarantees that the articulatory demands of nominally identical items are potentially highly variable and dependent on their sequential neighbors, closed stimulus sets such as those typically employed in serial recall experiments also ensure a high degree of predictability and regularity. This is especially true of phonologically similar items, in which articulatory variation is effectively restricted to the onset of each syllable (i.e., /p/, /v/, /t/, /dz/, /b/, /d/) and the rime is consistently /i:/. Under such conditions, articulations are likely to be highly conserved, irrespective of sequential order. Even in alternating sequences, conservation would play a role, since each dissimilar item is preceded by a common /i:/, again reducing the variability of coarticulation during rehearsal. Although speculative, this does suggest a possible mechanism by which item-like behavior might either emerge or be strongly reinforced during rehearsal. Such motoric factors, although significant in characterizing speech production, are usually marginalized, at best, in the study of short-term recall (but see Murray & Jones, 2002; Woodward, Macken, & Jones, 2008). Specifically, we identified the emergence of differential prosodic structures during rehearsal as a possible alternative influence on the patterns of transposition errors.

In both experiments, rehearsal gave rise to divergent stress patterns, which were readily and reliably attributed by a naïve third-party observer. In short—and consistent with our expectation—pairs rehearsal gave rise to an alternating and stress-initial (i.e., trochaic) structure, whilst triplets gave rise to a stress-initial and stress-terminal (i.e., cretic) structure. We propose that recall performance varies idiosyncratically as the interaction of stress and similarity plays out over the duration of extended rehearsal. Thus, in homogeneous sequences, terminal items are stressed in both trisyllables, but a recency effect is only obvious for the second trisyllable (Fig. 7). Most strikingly in the alternating sequences, a reversal in the phase of alternation does not simply invert the saw-tooth pattern of the serial position curve, but renders both stressed pair onsets and unstressed triplet-medial items equivalent in serial recall performance terms, independent of their nominal similarity (Fig. 3). We suggest instead that articulatory control mechanisms offer a plausible route for the emergence of transposition errors, particularly given the extended period of subvocalization inherent in the experimental design.

The idea that both metrical stress and sequential structure more generally influence transposition errors in serial recall chimes with similar views about their role in normal speech. Shattuck-Hufnagel (1992) proposed that the apparent bias toward interaction errors (e.g., transpositions) between initial segments in speech was confounded with the tendency toward stress-initial consonants in English speakers, with a greater likelihood of transposition when items (consonants) shared a common in-word position, even in the absence of common stress.

Whilst the present data are consistent with this general position, our interpretation differs in that we reject the primacy of a priori segment properties, emphasizing instead how stress may act as an integral component of articulatory control, such that stress becomes an intrinsic and differentiating component of the speech act for each articulatory instance at recall.

In terms of classical approaches to vSTM, articulatory contributions to the encoding and maintenance of written verbal material are uncontroversial (e.g., the phonological loop: Baddeley, 2012; Baddeley & Hitch, 1974), however such accounts predict no role for prosodic aspects of speech, regarding them as late-occurring production artifacts and as having no bearing on the representational stability of items in memory. However some recent studies have attempted to recast classical accounts of phonological storage—such as those based on the phonological loop—as a form of auditory-motor interface that serves to bind acoustic and articulatory representations (Buchsbaum et al., 2005; Jacquemot & Scott, 2006; Jones et al., 2006; Jones et al., 2004; and for a review, see Buchsbaum & D’Esposito, 2008). This broad approach conceptualizes phonological storage in terms of auditory–motor mapping rather than as the property of a storage system and provides a plausible environment in which sequence properties such as stress, pitch declination, rhythm can be represented as intrinsic aspects of organized auditory events, rather than as mere modifications to discrete phonological representations. In this context, stress is not simply a production artifact but forms part of the representational structure of to-be-remembered material.

Previous studies have illustrated how phonological similarity can be accounted for in terms of motor control processes and auditory perceptual organization and without recourse to abstract phonological knowledge (Jones et al., 2006; Jones et al., 2004; Maidment & Macken, 2012). The veracity of the more general claim—that language is subserved by discrete symbolic representation—has also been questioned (e.g., Port & Leary, 2005). Linguistic phenomena such as incomplete neutralization—in which nonidentical stops are neutralized to a flap by a vowel initial suffix (such as -*ing*) and yet remain partially, but not entirely, discriminable (Fox & Terbeek, 1977)—and the role of temporal ratios (between vowels and consonants) in defining perceptual boundaries between segments (Chen, 1970; Port, Salman, & Maeda, 1980) both argue against a static representational format and for a blurring of the distinction between the phonological and perceptual and motor control. Although inconclusive with respect to the precise mechanism of action, the present data add to this picture, confirming a specific role for temporally defined speech parameters in serial recall and supporting the more general case for the involvement of paralinguistic factors in tasks that are conventionally defined in primarily mnemonic and phonological terms.
